# Application of narrow band imaging and Lugol's iodine staining in screening for nasopharyngeal carcinoma

**DOI:** 10.1186/s12957-023-03258-5

**Published:** 2023-11-30

**Authors:** Fan Yang, Ning Huang, Xianming Chen, Maoxin Wang

**Affiliations:** Department of Otorhinolaryngology Head and Neck, Fuzong Clinical College of Fujian Medical University, the 900th Hospital of Joint Logistic Support Force of PLA, 156 West Second Ring North Road, Fuzhou, 0591 China

**Keywords:** Iodine staining, NBI, Endoscopy, Nasopharyngeal carcinoma

## Abstract

**Background:**

To investigate the diagnostic value of conventional white light endoscopy (WLE), narrow band imaging (NBI) endoscopy, and Lugol's iodine staining under WLE (endoscopic iodine staining) in the screening and early diagnosis of nasopharyngeal carcinoma.

**Methods:**

Patients with nasopharyngeal lesions requiring biopsy attending the Department of Otolaryngology Head and Neck Surgery in our hospital between January 2021 and April 2023 were included in this study. Before biopsy, all subjects underwent conventional WLE, NBI endoscopy, and endoscopic iodine staining. On WLE, according to nasopharyngeal lesion morphology and color, patients were diagnosed with nasopharyngeal carcinoma ( +) or chronic hyperplastic nasopharyngitis (-). On NBI endoscopy, according to nasopharyngeal lesion vascular morphology, patients with type V manifestations (nasopharyngeal carcinoma) were categorized as NBI ( +) and patients with type I-IV manifestations (chronic hyperplastic nasopharyngitis) were categorized as NBI (-). Endoscopic iodine staining (1.6% Lugol's iodine solution) was positive ( +) if the mucosal surface was brown with no white patches, or negative (-) if there was no or light brown staining of the mucosal surface. Patients were divided into 2 groups based on histopathological diagnosis: nasopharyngeal carcinoma or chronic hyperplastic nasopharyngitis. Endoscopic diagnoses were compared with histopathological findings. The diagnostic performance of WLE, NBI endoscopy and endoscopic iodine staining for nasopharyngeal carcinoma were determined.

**Results:**

This study included 159 patients. On histopathology, 29 patients were diagnosed with nasopharyngeal carcinoma, and 130 patients were diagnosed with chronic hyperplastic nasopharyngitis. There were no significant differences in the sensitivity, specificity, positive predictive value (PPV), negative predictive value (NPV), accuracy, and area under the receiver operating characteristic (ROC) curve (AUC) of conventional WLE, NBI endoscopy or endoscopic iodine staining for differentiating nasopharyngeal carcinoma and chronic hyperplastic nasopharyngitis. The diagnostic performance of the combination of conventional WLE, NBI endoscopy and endoscopic iodine staining was significantly improved compared to any procedure alone.

**Conclusions:**

Conventional WLE, NBI endoscopy or endoscopic iodine staining had good diagnostic performance for differentiating nasopharyngeal carcinoma and chronic hyperplastic nasopharyngitis. In particular, NBI endoscopy and endoscopic iodine staining alone or combined had clinical utility for identifying patients with nasopharyngeal lesions that are eligible for a watch-and-wait strategy.

## Background

Nasopharyngeal carcinoma is a malignant tumor of the head and neck with high incidence in Southeast Asia and south China [1, 2]. The gold standard for diagnosis of nasopharyngeal carcinoma is endoscopy and biopsy [3]. The prognosis of nasopharyngeal carcinoma is closely related to clinical stage. Many nasopharyngeal carcinomas can be cured if they are found and treated early; however, most patients present with advanced disease, with > 80% of patients presenting with lymph node metastases at the time of diagnosis [4]. There remains an unmet need to improve early diagnosis and treatment of nasopharyngeal carcinoma.

Discriminating nasopharyngeal carcinoma from chronic hyperplastic nasopharyngitis based on morphological features under white light endoscopy (WLE) is challenging. The diagnostic accuracy of WLE in the detection of nasopharyngeal carcinoma has high sensitivity and low specificity. Narrowband imaging (NBI) combined with WLE is increasingly used in clinical practice in the screening and diagnosis of head and neck malignant tumors [5, 6]. Endoscopic iodine staining, whereby mucosal lesions are stained with Lugol's iodine solution under endoscopy, may improve the diagnostic effectiveness of WLE and NBI endoscopy for mucosal lesions. Iodine staining has been applied in the diagnosis of oral mucosal tumors and esophageal lesions [7]. The objective of this study was to investigate the diagnostic value of conventional WLE, NBI endoscopy, and Lugol's iodine staining under WLE (endoscopic iodine staining) for screening and early diagnosis of nasopharyngeal carcinoma.

## Methods

### Patient population

Patients with nasopharyngeal lesions requiring biopsy attending the Department of Otolaryngology Head and Neck Surgery in our hospital between January 2021 and April 2023 were eligible for this study.

Inclusion criteria were 1) age ≥ 14 years with full capacity for civil conduct; 2) pharyngeal discomfort, blood in aspirated nasal discharge, tinnitus, ear pain, hearing loss, nasal congestion, neck swelling; 3) nasopharyngeal carcinoma indicated by indirect nasopharyngoscopy, computed tomography (CT) or magnetic resonance imaging (MRI) (clinical suspicion); 4) no history of iodine allergy; 5) no other lesions of the throat; 6) no acute and/or chronic respiratory diseases such as asthma, cough and sputum; 7) no history of cerebrovascular disease; 8) no history of hyperthyroidism; and 8) provided written informed consent to participate in the study.

Exclusion criteria were 1) nasopharyngeal carcinoma after radiotherapy; 2) aged < 14 years with adenoid hyperplasia; 3) severe heart, brain, liver and kidney dysfunction; 4) history of epilepsy; 5) lack of autonomy; 6) susceptible to allergies; 7) allergic to local anesthesia; or 8) severe deviation of the nasal septum and /or nasal stenosis that prevented passage of an endoscope.

This study was approved by the Hospital Ethics Committee.

### Procedures

All patients underwent conventional WLE, NBI endoscopy, and endoscopic iodine staining, followed by nasopharyngeal biopsy. Histopathological diagnosis was used as the gold standard.

Allergy to Lugol's iodine was determined by applying Lugol's iodine with cotton orally and interpreting staining of the sublingual mucosa of the anterior mouth after 1 min. Patients without mucosal edema and mucosal congestion were considered negative for iodine allergy. Patients with mucosal edema and mucosal congestion were considered positive for iodine allergy.

Bupivacaine hydrochloride gel was injected into the nasal cavity and throat for local anesthesia. Patients were comfortably seated, and a flexible nasopharyngoscope (Olympus, IpX7) was inserted through the anterior nostril. Routine examination of the nasal cavity was performed with the flexible nasopharyngoscope. Nasopharyngeal lesions were examined with conventional WLE. Tumor morphology was revealed using NBI endoscopy. For endoscopic iodine staining, the nasopharyngeal mucus was removed through endoscopy, maintaining the surface of the lesion intact. 1.5 mL of a 1.6% Lugol's iodine solution was sprayed from the top of the nasopharynx to avoid mucosal irritation. After spraying, the patients were instructed to swallow several times to prevent coughing. Staining was interpreted under WLE after 1 min. Patients were observed for 30 min after iodine staining to ensure there were no adverse effects. Tumor biopsy was performed with nasopharyngeal biopsy forceps under oral endoscopy. Biopsy specimens were fixed in 10% formalin and examined in the Pathology Department. After biopsy, patients were instructed to gargle with plenty of water and rinse the nasal cavity with normal saline.

### Outcomes

WLE and NB images were examined by 2 senior and experienced attending physicians in the Department of Otolaryngology Head and Neck Surgery who were blinded to patients’ medical history and physical examination. Nasopharyngeal biopsies were examined by 2 senior and experienced attending physicians and reviewed by 1 associate senior physician in the Pathology Department who were blinded to patients’ medical history, physical examination, and findings on WLE, NBI endoscopy and endoscopic iodine staining.

On WLE, according to nasopharyngeal lesion morphology and color, patients were diagnosed with nasopharyngeal carcinoma ( +) or chronic hyperplastic nasopharyngitis (-). On NBI endoscopy, according to nasopharyngeal lesion vascular morphology, patients with type V manifestations (nasopharyngeal carcinoma) were categorized as NBI ( +) and patients with type I-IV manifestations (chronic hyperplastic nasopharyngitis) were categorized as NBI (-) [8–10] Endoscopic iodine staining was positive ( +) if the mucosal surface was brown with no white patches or negative (-) if there was no or light brown staining of the mucosal surface.

Patients were divided into 2 groups based on histopathological diagnosis: nasopharyngeal carcinoma or chronic hyperplastic nasopharyngitis.

### Statistical analysis

Statistical analyses were conducted with SPSS v27. The diagnostic performance of conventional WLE, NBI endoscopy, endoscopic iodine staining, or the combination of conventional WLE, NBI endoscopy and endoscopic iodine staining modeled using logistic regression, for differentiating nasopharyngeal carcinoma and chronic hyperplastic nasopharyngitis were determined and compared by calculating sensitivity, specificity, positive predictive value (PPV), negative predictive value (NPV) and accuracy, and using receiver operating characteristic (ROC) curve analysis. *p* < 0.05 was considered statistically significant.

## Results

This study included 178 patients. 19 patients were excluded from the analyses due to excessive bleeding in nasopharyngeal lesions before endoscopic iodine staining, findings on NBI endoscopy and endoscopic iodine staining could not be interpreted, and/or the presence of other types of nasopharyngeal malignancies. Finally, 159 patients (Table 1) (*n* = 89 males; *n* = 70 females) with a mean age of 42.16 years were included in the study. Epstein-Barr virus (EBV) is associated with nasopharyngeal carcinoma, and the detection of EBV-related markers has been routinely used in the diagnosis of nasopharyngeal carcinoma. In this study, all 159 patients were tested for EBV-DNA in peripheral plasma after nasopharyngoscopy. EBV-DNA was detected in 96.55% of patients with nasopharyngeal carcinoma and 21.31% of patients with chronic hyperplastic nasopharyngitis. The sensitivity, specificity, PPV, NPV, accuracy, and area under the ROC curve (AUC) for EBV-DNA for differentiating nasopharyngeal carcinoma and chronic hyperplastic nasopharyngitis were as follows: sensitivity 96.552%, specificity 87.692%, PPV 63.636%, NPV 99.130%, accuracy 89.308%and AUC 0.921, 95%CI 0.868–0.958.

**Table 1:** The clinical characteristics of patients with NPC and controls

		Nasopharyngeal carcinoma (*n* = 29)	Chronic hyperplastic nasopharyngitis (*n* = 130)
Mean age(years)		48.10	41.60
Sex	Male	17	71
	Female	12	59
Smoking	Yes	10	21
	No	19	109
Drinking Alcohol	Yes	8	19
	No	21	111
Hypertension	Yes	8	23
	No	21	103
Diabetes	Yes	7	5
	No	22	125
EBV-DNA	Positive	28	16
	Negative	1	114

*Drinking alcohol* Drinking on a continuous daily basis > 50 ml

Note: EBV-DNA positive: the copy number of EBV-DNA in peripheral plasma was > 1000

On histopathology, 29 patients were diagnosed with nasopharyngeal carcinoma, and 130 patients were diagnosed with chronic hyperplastic nasopharyngitis (Table 2).

**Table 2:** Comparison of findings on endoscopic screening and histopathology

Endoscope mode	Endoscopic screening results	Histopathological results		Total	χ^2^	*p*
		Chronic hyperplastic nasopharyngitis (*n* = 130)	NPC (*n* = 29)			
WLE	Chronic nasopharyngitis	98	8	106	24.377	< 0.01
	NPC	32	21	53		
NBI	Chronic nasopharyngitis	111	10	121	33.778	< 0.01
	NPC	19	19	38		
Iodine staining	Chronic nasopharyngitis	106	5	111	19.392	< 0.01
	NPC	24	24	48		
Combined prediction	Chronic nasopharyngitis	127	12	139	68.901	< 0.01
	NPC	3	17	20		

*NPC* Nasopharyngeal carcinoma, *WLE* White light endoscopy, *NBI* Narrow band imaging

**Fig. 1:**
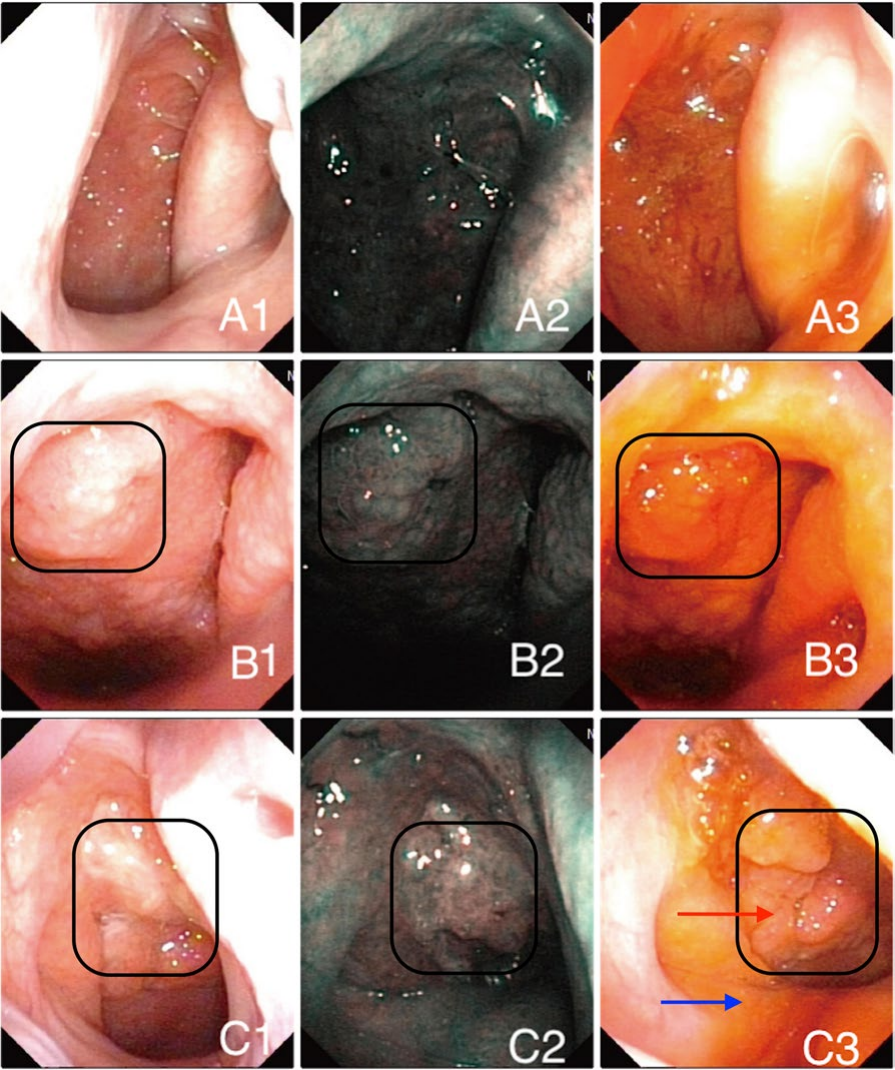
**A** Healthy control, normal nasopharynx: 1 WLE, 2 NBI endoscopy, 3 Endoscopic iodine staining. **B** Chronic hyperplastic nasopharyngitis: 1 WLE, 2 NBI endoscopy showing cobblestone features, 3 Endoscopic iodine staining. Uniform staining was observed. **C** Nasopharyngeal carcinoma: 1 WLE, 2 NBI endoscopy, 3 Endoscopic iodine staining. The tumor was more obvious than the surrounding normal tissue and inflammatory tissue. The red arrow shows an unstained area after exposure to Lugol's iodine solution, and the blue arrow shows staining with Lugol's iodine solution on the back of the soft palate. Black box: observation area

Figure 1 shows representative images of a normal nasopharynx (Fig. A), chronic hyperplastic nasopharyngitis, and nasopharyngeal carcinoma under WLE, NBI endoscopy and endoscopic iodine staining. The observation area incorporated abnormal protruding tissue in the nasopharynx, the torus, the back of the soft palate, the transition from normal mucosa to proliferative tissue, and the contralateral nasopharynx. The probability of inflammatory hyperplasia was greater in patients with cobblestone-like changes under NBI endoscopy (Fig. B2) and uniform Lugol's iodine staining (Fig. B3). Nasopharyngeal carcinoma or other malignant tumors should be highly suspected in patients with hyperplastic and tortuous surface blood vessels under NBI endoscopy **(**Fig. C2**)**, and areas exposed to Lugol's iodine solution that are not colored, or the color is light compared with the surrounding normal tissue** (**Fig. C3**).**

**Fig. 2:**
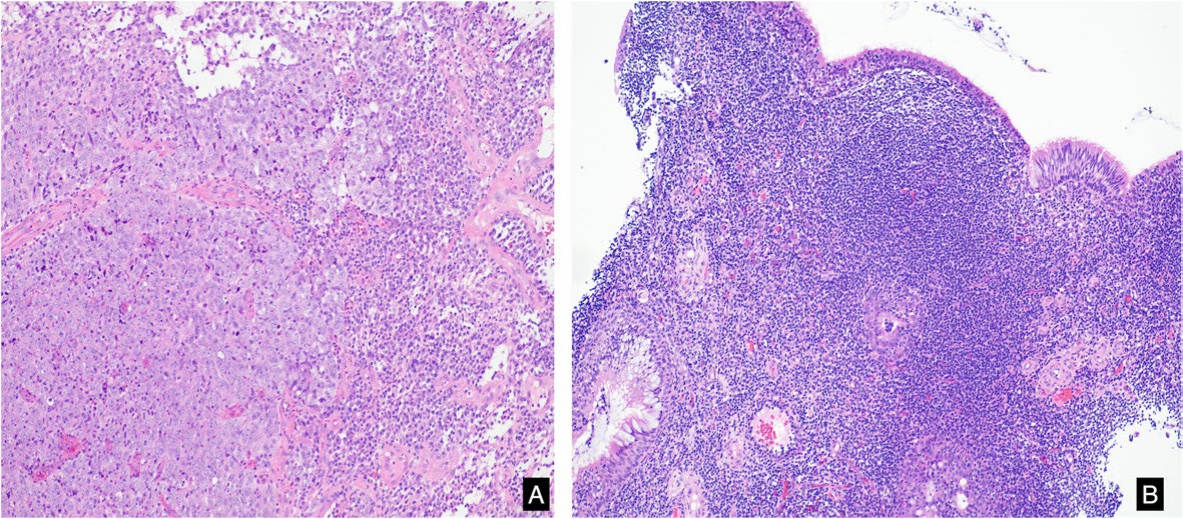
**A:** Nasopharyngeal carcinoma stained with HE (× 100) **B**: Chronic hyperplastic nasopharyngitis stained with HE (× 100)

**Fig. 3:**
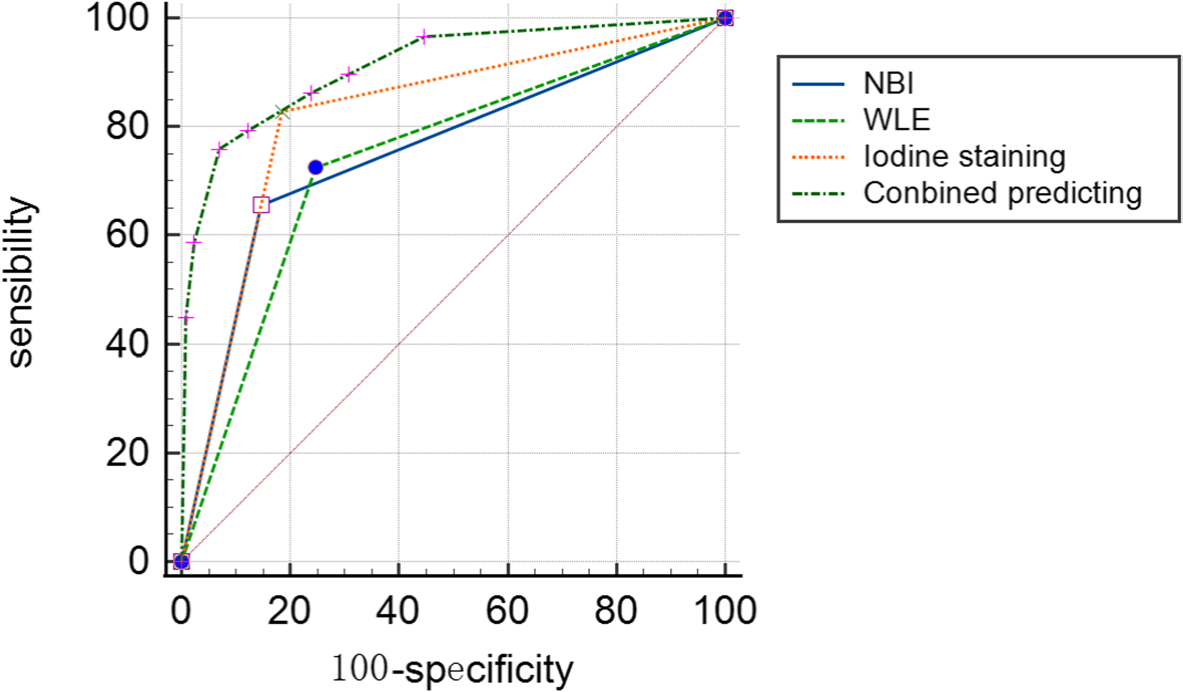
Comparison of conventional WLE, endoscopic iodine staining, NBI and combined prediction AUC curve

The sensitivity, specificity, PPV, NPV, accuracy, and AUC for conventional WLE, NBI endoscopy or endoscopic iodine staining for differentiating nasopharyngeal carcinoma and chronic hyperplastic nasopharyngitis were as follows: conventional WLE, sensitivity 72.414%, specificity 75.385%, PPV 39.623%, NPV 92.453%, accuracy 74.843%. AUC 0.739, 95%CI 0.664–0.805; NBI endoscopy, sensitivity 65.517%, specificity 85.385%, PPV 50.000%, NPV 91.736%, accuracy 81.761%, AUC 0.755, 95%CI 0.680–0.819; and endoscopic iodine staining, sensitivity 82.759%, specificity 81.538%, PPV 50.000%, NPV 95.495%, accuracy 81.761%, AUC 0.821, 95%CI 0.753–0.878. There were no significant differences in the diagnostic performance of conventional WLE, NBI endoscopy or endoscopic iodine staining (Table 3).

**Table 3:** Sensitivity and specificity of WLE, NBI endoscopy and endoscopic iodine staining compared with histopathological results

	Sensitivity (%)	Specificity (%)	PPV (%)	NPV (%)	AUC	95%CI	*p*	Accuracy (%)
WLE	72.414	75.385	39.623	92.453	0.739	0.664–0.805	< 0.01	74.843
NBI	65.517	85.385	50.000	91.376	0.755	0.680–0.819	< 0.01	81.761
Iodine staining	82.759	81.538	50.000	95.495	0.821	0.753–0.878	< 0.01	81.761
Combined prediction	85.000	91.367	58.621	97.692	0.912	0.857–0.951	< 0.01	90.566

*WLE* White light endoscopy, *NBI* Narrow band imaging, *PPV* Positive predictive value, *NPV* Negative predictive value, *AUC* Area under curve

Note: *p* < 0.05 for conventional WLE, endoscopic iodine staining, or NBI vs. combined prediction

The sensitivity, specificity, PPV, NPV, accuracy, and AUC for the combination of conventional WLE, NBI endoscopy and endoscopic iodine staining for differentiating nasopharyngeal carcinoma and chronic hyperplastic nasopharyngitis, modeled using logistic regression, were as follows: sensitivity 85.000%, specificity 91.376%, PPV 58.621%, NPV 97.692%, accuracy 90.566%, AUC 0.921 95%CI 0.857–0.951. The diagnostic performance of the combination of conventional WLE, NBI endoscopy and endoscopic iodine staining was significantly improved compared to any method alone (Table 3).

On binary regression analysis the hazard ratio estimates for nasopharyngeal carcinoma (+ vs. -) on conventional WLE, NBI endoscopy or endoscopic iodine staining were 4.797, 7.552, or 17.221, respectively (Table 4).

**Table 4:** Regression analysis of three screening methods

	B	SE	Wald	OR	95%CI		*p*
WLE	1.568	0.58	7.313	4.797	1.54	14.948	0.007
NBI	2.022	0.588	11.818	7.552	2.385	23.913	< 0.01
Iodine staining	2.846	0.617	21.296	17.221	5.142	57.682	< 0.01
Constant	-10.908	1.762	38.307	0			0.00

*WLE* White light endoscopy, *NBI* Narrow band imaging

## Discussion

The present study investigated the diagnostic value of conventional WLE, NBI endoscopy and Lugol's iodine staining under WLE (endoscopic iodine staining) in the screening and early diagnosis of nasopharyngeal carcinoma. There were no significant differences in the diagnostic performance of conventional WLE, NBI endoscopy or endoscopic iodine staining for differentiating nasopharyngeal carcinoma and chronic hyperplastic nasopharyngitis. The diagnostic performance of the combination of conventional WLE, NBI endoscopy and endoscopic iodine staining was significantly improved compared to any method alone.

Current screening methods, including EBV antibody test, CT/MRI, positron emission tomography scan, conventional white light nasopharyngoscopy, and NBI endoscopy, can improve early diagnosis of nasopharyngeal carcinoma. Conventional WLE is commonly used in endoscopic examinations of nasopharyngeal carcinoma. However, conventional WLE does not detect subtle changes in morphology or mucosal blood vessels in the nasopharyngeal region, and WLE cannot differentiate between nasopharyngeal carcinoma and chronic nasopharyngeal mucosal inflammation based on color and morphologic (apophysis or anabrosis) abnormalities [10]. This can lead to misdiagnosis and missed diagnosis of nasopharyngeal carcinoma.

NBI is an endoscopic technique that uses filters to illuminate the mucosa with light from selected bands (415 nm, blue light; 540 nm, green light) of the optical spectrum. The filtered light preferentially enhances the mucosal surface and the network of superficial blood vessels [11]. NBI endoscopy can identify intrapapillary capillary loops (IPCLs) in stratified epithelium, which are destroyed and replaced by irregular neo-tumor vasculature. NBI endoscopy is limited by a number of factors, including the presence of mucus, blood and keratin that can significantly interfere with the interpretation of NBI features [12]. NBI endoscopy is widely used in the diagnosis of head, neck, throat and hypopharyngeal lesions, and there are multiple NBI endoscopic classifications based on vascular changes for different organs [13]. A NBI classification of nasopharyngeal mucosal microvessels has been developed to facilitate differential diagnosis of benign and malignant lesions of the nasopharyngeal region (Types I-IV benign, Type V, malignant). Previous studies based on this classification suggest the sensitivity and specificity of NBI endoscopy for nasopharyngeal lesions are higher than conventional WLE [14]. The sensitivity, specificity, accuracy, PPV, and NPV for distinguishing nonmalignant from malignant lesions were 97.4%, 84.6%, 92.7%, 91.6%, and 95.1%, respectively, for patients with pharyngolaryngeal lesions, 98% of histologically malignant lesions corresponded to a type V pattern, and 84.8% of non-neoplastic lesions corresponded to a type I to IV pattern [15]. The characteristics of nasopharyngeal carcinoma under NBI endoscopy mainly appeared as a type V pattern (79.5%, 167/210), and the sensitivity, specificity, PPV, and NPV of type V in the diagnosis of nasopharyngeal carcinoma were 79.5%, 91.3%, 96.0%, and 62.9%, respectively, in patients with a suspected nasopharyngeal tumor. NBI endoscopy significantly improved the detection of superficial lesions (χ^2 ^= 12.789, *p* < 0.0001) compared to WLE [16]. The sensitivity and NPV of NBI endoscopy for nasopharyngeal carcinoma screening were significantly higher than those of WLE (93.9% vs 71.2%, *P* = 0.001; and 98.1% vs 91.7%, *P* = 0.003; respectively) in consecutive patients at high risk for nasopharyngeal carcinoma. The presence of superficial, distorted, irregularly shaped microvessels on NBI endoscopy indicated malignant lesions [17].

Endoscopic iodine staining with Lugol's iodine is widely used in head and neck endoscopy and can assist in delineating normal and abnormal margins, informing endoscopic resection [18]. Lugol's iodine stains glycogen in normal squamous epithelium, which appears brown under WLE. In glycogen depleted epithelium such as dysplasia, the mucosa appears yellow or unstained [19]. Knowledge of head and neck anatomy is necessary when interpreting Lugol’s staining of nasopharyngeal lesions. The attached gingivae and hard palate, which are heavily keratinized, and the respiratory mucosa, which does not contain glycogen, do not stain with Lugol’s iodine [20]. The glycogen containing cells lining the oral cavity and oropharynx do stain [21] Endoscopic iodine staining is limited by the presence of blood and allergic reactions to Lugol’s iodine, including laryngospasm, bronchospasm, and cardiac arrest. High concentrations of Lugol’s iodine may result in large amounts of free iodine, which can cause mucosal damage [20]. Spraying Lugol’s iodine solution in the pharynx is associated with heartburn, severe discomfort, and risk of aspiration [22]. In the present study, according to previously published literature, 1.5 ml of a 1.6% iodine solution was sprayed from the top of the nasopharynx [7] with few adverse effects. Timing of the interpretation of endoscopic iodine staining is subjective and requires a certain amount of clinical experience. In patients with suspected esophageal cancer, the appearance of a color change (designated as a pink-color sign [PCS]) within 1 min after Lugol’s iodine staining had a higher diagnostic performance (sensitivity, 90.2%; specificity, 82.3%; diagnostic accordance rate of 88.6%), than appearance of a PCS at 2 min (sensitivity, 84.1%; specificity, 72.7%; diagnostic accordance rate of 79.7%) [23]. The rapid appearance of PCS seems to indicate a high degree of epithelial destruction. The timing of the appearance of PCS was significantly and independently associated with high-grade intraepithelial neoplasia/invasive cancer. In the present study, patients were seated and excess iodine solution was removed by coughing or blowing the nose. Reexamination was performed 1 min after spraying with Lugol’s iodine solution.

A previous report showed that NBI endoscopy combined with endoscopic iodine staining may be beneficial for the diagnosis of esophageal cancer and precancerous lesions. Similarly, our findings suggest the diagnostic performance of the combination of conventional WLE, NBI endoscopy and endoscopic iodine staining for discriminating between nasopharyngeal carcinoma and chronic hyperplastic nasopharyngitis was significantly improved compared to any method alone. Importantly, NBI endoscopy and endoscopic iodine staining alone or combined had a high NPV for nasopharyngeal carcinoma, implying these methods may identify patients with a high probability of no clinically significant cancer that may avoid unnecessary biopsy and surgeries. This indicates that NBI endoscopy and endoscopic iodine staining may have clinical utility for selecting patients with nasopharyngeal lesions that are eligible for a watch-and-wait strategy.

## Conclusions

In conclusion, despite the limitations of a small sample size of patients enrolled from a single medical center, this study shows that NBI endoscopy and endoscopic iodine (1.6% iodine; 1 min interval to examination) staining may facilitate personalized management of patients with nasopharyngeal lesions.

## Data Availability

The datasets used and/or analyzed during the current study are available from the corresponding author on reasonable request.

## Abbreviations

WLE: White light endoscopy
NBI: Narrow band imaging
PCS: Designated as a pink-color sign
PPV: Positive predictive value
NPV: Negative predictive value
ROC: The receiver operating characteristic curve
AUC: Area Under Curve
IPCLs: Identify intrapapillary capillary loops
NPC: Nasopharyngeal carcinoma
